# Letter to the editor concerning “A propensity-matched study of patients with symptomatic lumbar spinal stenosis opting for surgery versus not” by Friis Pedersen et al.

**DOI:** 10.1016/j.bas.2024.102871

**Published:** 2024-07-14

**Authors:** Rikke K. Jensen, James J. Young, Jan Hartvigsen

**Affiliations:** Center for Muscle and Joint Health, Department of Sports Science and Clinical Biomechanics, University of Southern Denmark, Odense, Denmark; Chiropractic Knowledge Hub, University of Southern Denmark, Odense, Denmark; Center for Muscle and Joint Health, Department of Sports Science and Clinical Biomechanics, University of Southern Denmark, Odense, Denmark; Schroeder Arthritis Institute, Krembil Research Institute, University Health Network, Toronto, Canada; Center for Muscle and Joint Health, Department of Sports Science and Clinical Biomechanics, University of Southern Denmark, Odense, Denmark; Chiropractic Knowledge Hub, University of Southern Denmark, Odense, Denmark

To the Editor,

Surgical versus non-surgical care for people with lumbar spinal stenosis (LSS) is a hot topic everywhere because of aging populations. Therefore, we read with interest the study by Friis Pedersen et al. (Friis Pedersen et al., 2024) dealing with a propensity matched comparison of outcomes after surgery or no surgery in patients diagnosed with LSS. Unfortunately, the paper suffers from serious methodological flaws and the conclusions are not supported by the data and analysis presented.

First, there is no standardised, or at least classified, description of the non-surgical care, which prevents a comparison between the groups. Based on the data presented, it is not possible to determine whether the observed outcomes were due to the superiority of surgical treatment or variations in the quality and appropriateness of the non-surgical interventions.

Second, the cohort design is inherently limited by its observational nature, which is less able to control for confounding variables than randomised controlled trials (RCT). Nonetheless, studies based on propensity score matching can be informative if they include relevant baseline variables and employ appropriate methods for assessing balance of these variables between treatment groups (Austin, 2011; Cote et al., 2024; Hernan and Robins, 2016; VanderWeele and Ding, 2017). Unfortunately, neither is the case in the study by Friis Pedersen et al.

1) The study's propensity score model omits several key confounders (baseline variables) such as comorbidities and psychosocial socio-psychological factors, which increases the risk of unmeasured confounding and potentially biases the outcome analysis.
2) After matching, instead of using recommended methods like calculating standardised differences or plotting propensity score distributions to assess balance, the authors calculated mean or proportion differences with associated p-values, which is inappropriate because it fails to accurately indicate balance on important baseline confounders (Austin, 2011).
3) After performing balance diagnostics, treatment outcome differences should typically be estimated using RCT-like methods, adjusting for unbalanced baseline variables (Cote et al., 2024; Hernan and Robins, 2016). Friis Pedersen et al., however, simply compared mean or proportion differences at the 1-year follow-up without accounting for differences between the matched groups.
4) The final step in using observational data to estimate treatment effectiveness involves conducting a sensitivity analysis, such as calculating the E-value to estimate the potential impact of unmeasured confounding (Cote et al., 2024; Hernan and Robins, 2016; VanderWeele and Ding, 2017). Here, the authors failed to perform such an analysis, leaving concerns about significant unmeasured confounding unaddressed.

Finally, there is no information about why patients were assigned to non-surgical treatments after being assessed for surgery. It is crucial to understand which factors influence decisions about whether to operate or not (Chen et al., 2010; Jensen et al., 2023), as these may include variables that could independently affect outcomes (Aalto et al., 2006).

Both individually and taken together, these methodological flaws and lack of treatment data significantly undermine the validity of the conclusion that offering most patients spinal decompression is the best option in the treatment of patients with LSS.

## Declaration of competing interest

The authors declare that they have no known competing financial interests or personal relationships that could have appeared to influence the work reported in this paper.

## References

[bib1] Aalto T.J., Malmivaara A., Kovacs F., Herno A., Alen M., Salmi L. (2006). Preoperative predictors for postoperative clinical outcome in lumbar spinal stenosis: systematic review. Spine.

[bib2] Austin P.C. (2011). An introduction to propensity score methods for reducing the effects of confounding in observational studies. Multivariate Behav. Res..

[bib3] Chen E., Tong K.B., Laouri M. (2010). Surgical treatment patterns among Medicare beneficiaries newly diagnosed with lumbar spinal stenosis. Spine J..

[bib4] Cote P., Negrini S., Donzelli S., Kiekens C., Arienti C., Ceravolo M.G. (2024). Introduction to target trial emulation in rehabilitation: a systematic approach to emulate a randomized controlled trial using observational data. Eur. J. Phys. Rehabil. Med..

[bib5] Friis Pedersen C., Eiskjaer S., Osterheden Andersen M., Yacat Carreon L., Doering P. (2024). A propensity-matched study of patients with symptomatic lumbar spinal stenosis opting for surgery versus not. Brain Spine.

[bib6] Hernan M.A., Robins J.M. (2016). Using big data to emulate a target trial when a randomized trial is not available. Am. J. Epidemiol..

[bib7] Jensen R.K., Skovsgaard C.V., Ziegler D.S., Schiottz-Christensen B., Mieritz R.M., Andresen A.K. (2023). Surgical trends and regional variation in Danish patients diagnosed with lumbar spinal stenosis between 2002 and 2018: a retrospective registry-based study of 83,783 patients. BMC Health Serv. Res..

[bib8] VanderWeele T.J., Ding P. (2017). Sensitivity analysis in observational research: introducing the E-value. Ann. Intern. Med..

